# Multiple Myeloma With Renal Pathological Findings of Both Light Chain-Only Variant of Proliferative Glomerulonephritis With Monoclonal Immunoglobulin Deposits (PGNMID-LC) and Light Chain Deposition Disease (LCDD)

**DOI:** 10.7759/cureus.89788

**Published:** 2025-08-11

**Authors:** Kuya Okazaki, Akira Shimizu, Masanori Sakakima

**Affiliations:** 1 Internal Medicine, Hamamatsu University School of Medicine, Hamamatsu, JPN; 2 Analytic Human Pathology, Graduate School of Medicine, Nippon Medical School, Tokyo, JPN; 3 Internal Medicine, Fujinomiya City General Hospital, Fujinomiya City, JPN

**Keywords:** light chain deposition disease (lcdd), light chain-only variant of proliferative glomerulonephritis with monoclonal immunoglobulin deposits (pgnmid-lc), membranoproliferative glomerulonephritis (mpgn), multiple myeloma, proliferative glomerulonephritis with monoclonal immunoglobulin deposits (pgnmid)

## Abstract

Light chain-only variant of proliferative glomerulonephritis with monoclonal immunoglobulin deposits (PGNMID-LC) and light chain deposition disease (LCDD) are both renal disorders caused by the overproduction of monoclonal immunoglobulin light chains and their deposition in renal tissues. However, the renal pathological features of these two entities are characteristically distinct. We report a rare case of multiple myeloma presenting with renal pathology exhibiting overlapping features of both PGNMID-LC and LCDD. A 54-year-old woman presented with nephrotic syndrome. Renal biopsy revealed glomerular lesions resembling type III membranoproliferative glomerulonephritis on light microscopy. Immunofluorescence microscopy demonstrated κ light chain deposition along the glomerular capillary walls as well as in the mesangial areas, Bowman’s capsule, and tubular basement membranes. C3 was also deposited in the glomeruli, while IgG and λ light chain staining were negative. Electron microscopy revealed dense deposits in the mesangial, subepithelial, and subendothelial areas, along with powdery deposits within the glomerular basement membrane, tubular basement membranes, and Bowman’s capsule. Monoclonal κ light chains were detected in both serum and urine, and bone marrow examination showed 30.6% monoclonal plasma cells. The patient was diagnosed with multiple myeloma-associated kidney disease due to monoclonal light chain deposition and was treated with bortezomib and dexamethasone, resulting in improved renal function and a marked reduction in proteinuria. This case highlights a rare pathological overlap between PGNMID-LC and LCDD, underscoring the importance of comprehensive renal pathological evaluation in patients with monoclonal gammopathies.

## Introduction

Paraproteins, monoclonal immunoglobulins or their fragments produced by abnormal plasma cells or B cells, can deposit in the kidney and cause a variety of renal lesions [1]. These lesions are classified into several types based on their pathological characteristics [2], and among those characterized by light chain-only deposits are light chain deposition disease (LCDD) and the light chain-only variant of proliferative glomerulonephritis with monoclonal immunoglobulin deposits (PGNMID-LC). LCDD typically presents with nodular glomerulosclerosis and light chain deposits in the glomerular basement membrane (GBM) and tubular basement membrane (TBM) [3], whereas PGNMID-LC is associated with membranoproliferative glomerulonephritis (MPGN), like glomerular lesions and light chain deposition within the glomeruli [4]. Although both entities are thought to result from the deposition of monoclonal free light chains, they are considered distinct diseases. Reports describing cases that combine features of both entities are extremely rare.

In this report, we describe a case of multiple myeloma with rare renal pathological findings that exhibited both MPGN type III-like glomerular lesions and monoclonal free light chain deposition in the GBM, TBM, and Bowman’s capsule, features consistent with a combination of PGNMID-LC and LCDD.

## Case presentation

A 54-year-old woman with no significant past medical history was referred to our hospital for further evaluation after a routine health checkup revealed hypoalbuminemia (serum albumin: 1.6 g/dL) and proteinuria (3+ on urine dipstick), approximately one month following the onset of edema. Her vital signs were as follows: body temperature of 36.4°C, blood pressure of 120/60 mmHg, pulse rate of 91 bpm, and oxygen saturation of 99% on room air. Physical examination was unremarkable except for bilateral pitting edema in the lower extremities extending to below the knees. The patient reported a 3 kg weight gain from her baseline weight of 63 kg over the past month, coinciding with the onset of edema. She denied shortness of breath, orthopnea, or paroxysmal nocturnal dyspnea. Laboratory data at admission are summarized in Table 1. She presented with nephrotic syndrome, characterized by hypoalbuminemia, severe proteinuria (24-hour urine protein excretion: 3.9 g/day), and edema, despite normal serum creatinine levels. Serologic testing revealed no evidence of hepatitis B or C virus infection, collagen vascular disease, or cryoglobulinemia. Serum and urine immunoelectrophoresis detected free κ light chains. The serum-free κ light chain level was markedly elevated at 3220 mg/L (reference range: 3.3-19.4 mg/L), while the serum-free λ light chain level was 17 mg/L (reference range: 5.71-26.3 mg/L), resulting in a κ/λ ratio of 182, indicating a marked monoclonal κ light chain predominance.

**Table 1:** Laboratory data on admission.

Test	Patient value	Reference range
Complete blood cell count		
White blood cells	4200	3500-8500/µL
Hemoglobin	12.2	11.0-15.0 g/dL
Platelets	32	15-35 ×104/µL
Blood chemistry		
Aspartate aminotransferase	25	10-40 IU/L
Alanine aminotransferase	23	5-40 IU/L
Alkaline phosphatase	79	100-325 IU/L
Total protein	4.1	6.7-8.3 g/dL
Albumin	1.6	3.8-5.3 g/dL
Triglycerides	134	50-149 mg/dL
Total cholesterol	256	120-219 mg/dL
Low-density lipoprotein cholesterol	229	<120 mg/dL
Serum urea nitrogen	15.7	8-22 mg/dL
Creatinine	0.54	0.40-1.10 mg/dL
Estimated glomerular filtration rate	89.5	>90 mL/min/1.73 m2
Uric acid	5.4	2.5-7.0 mg/dL
Sodium	143	136-147 mEq/L
Potassium	4.4	3.5-5.0 mEq/L
Chloride	111	98-107 mEq/L
Calcium	7.8	8.7-10.3 mg/dL
Inorganic phosphate	3.8	2.5-4.5 mg/dL
C-reactive protein	0.02	<0.30 mg/dL
Hemoglobin A1c	5.6	<6.5%
Blood glucose	121	70-109 mg/dL
Immunoglobulin G	335	870-1700 mg/dL
Immunoglobulin A	134	110-410 mg/dL
Immunoglobulin M	26	35-220 mg/dL
C3	97	86-160 mg/dL
C4	58	17-45 mg/dL
Hemolytic complement activity	56	25-48 CH50/mL
Urinalysis		
pH	7.1	5.0-8.0
Protein	5.89	<0.15 g/g·creatinine
Red blood cells	5-6	0-2/high power field
Creatinine	61	50-200 mg/dL

**Figure 1:**
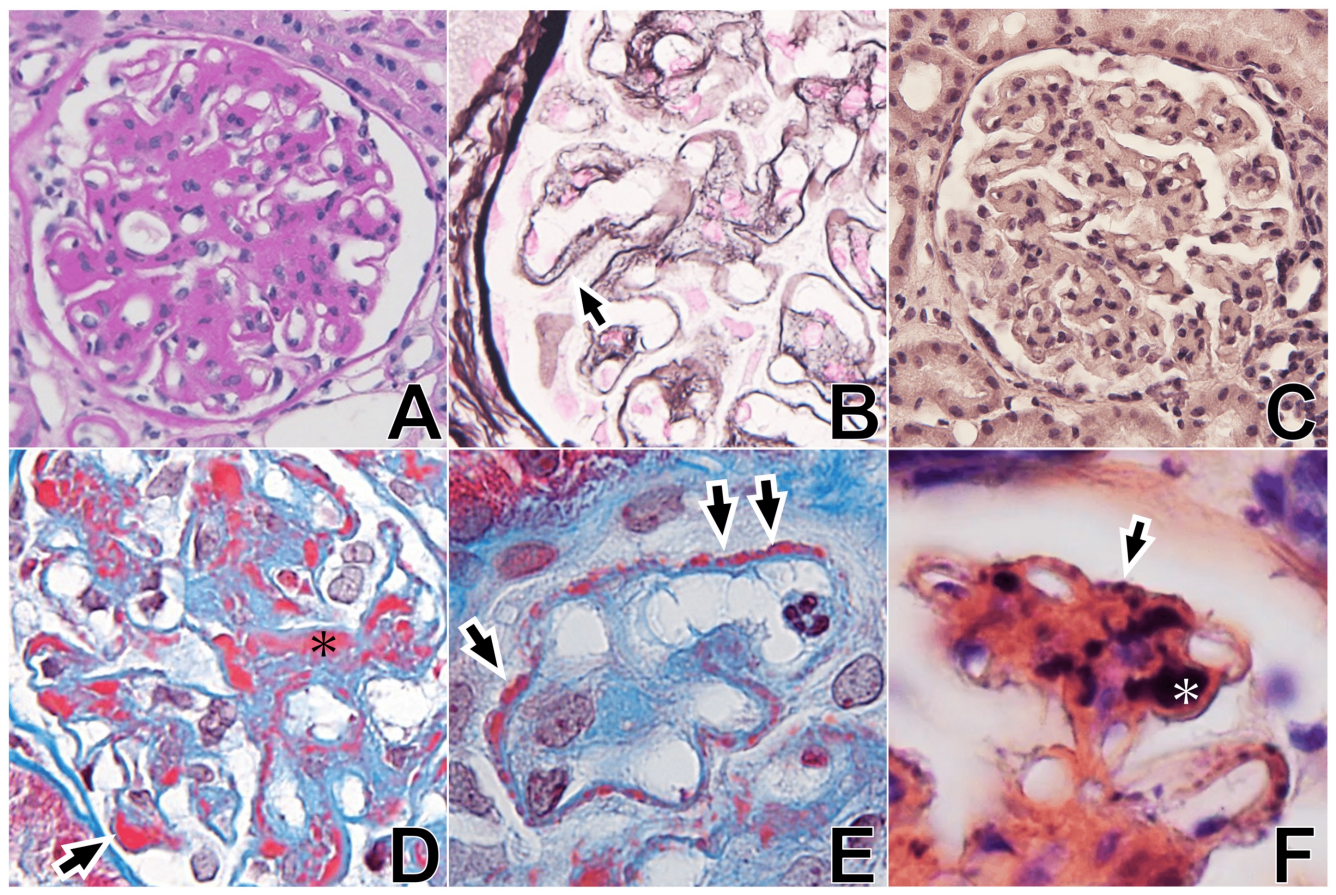
Light microscopic findings. (A) Periodic acid–Schiff staining of the renal biopsy specimen shows increased mesangial matrix, mesangial cell proliferation, diffuse thickening of the capillary walls, and lobular appearance. Original magnification: ×400. (B) Periodic acid methenamine silver staining demonstrates duplication of the glomerular capillary walls (arrow). Original magnification: ×400. (C) Direct fast scarlet staining was negative for amyloid. Original magnification: ×400. (D, E) Masson's trichrome staining reveals red deposits in the subendothelial and subepithelial regions of the glomerular capillary loops (arrows), and in the mesangial areas (asterisk in D). Original magnification: ×400. (F) Phosphotungstic acid–hematoxylin staining shows positive signals in the subendothelial (asterisk) and subepithelial (arrow) regions of the glomerular capillary walls, as well as in the mesangium. Original magnification: ×400.

Renal biopsy was performed the day after admission. The specimen included 32 glomeruli, of which one exhibited global sclerosis. Periodic acid-Schiff (PAS) staining showed mesangial cell proliferation, expansion of the mesangial matrix, and lobular segmentation of capillary loops (Figure 1A). Periodic acid methenamine silver (PAM) staining revealed thickening of the glomerular capillary walls with double contours (Figure 1B). Direct fast scarlet staining was negative (Figure 1C). Masson's trichrome staining demonstrated red deposits in the mesangial, subepithelial, and subendothelial regions of the glomerular basement membrane, consistent with type III MPGN-like lesions (Figures 1D, 1E). Phosphotungstic acid-hematoxylin (PTAH) staining was positive in the mesangial areas and subendothelial and subepithelial regions (Figure 1F).

**Figure 2:**
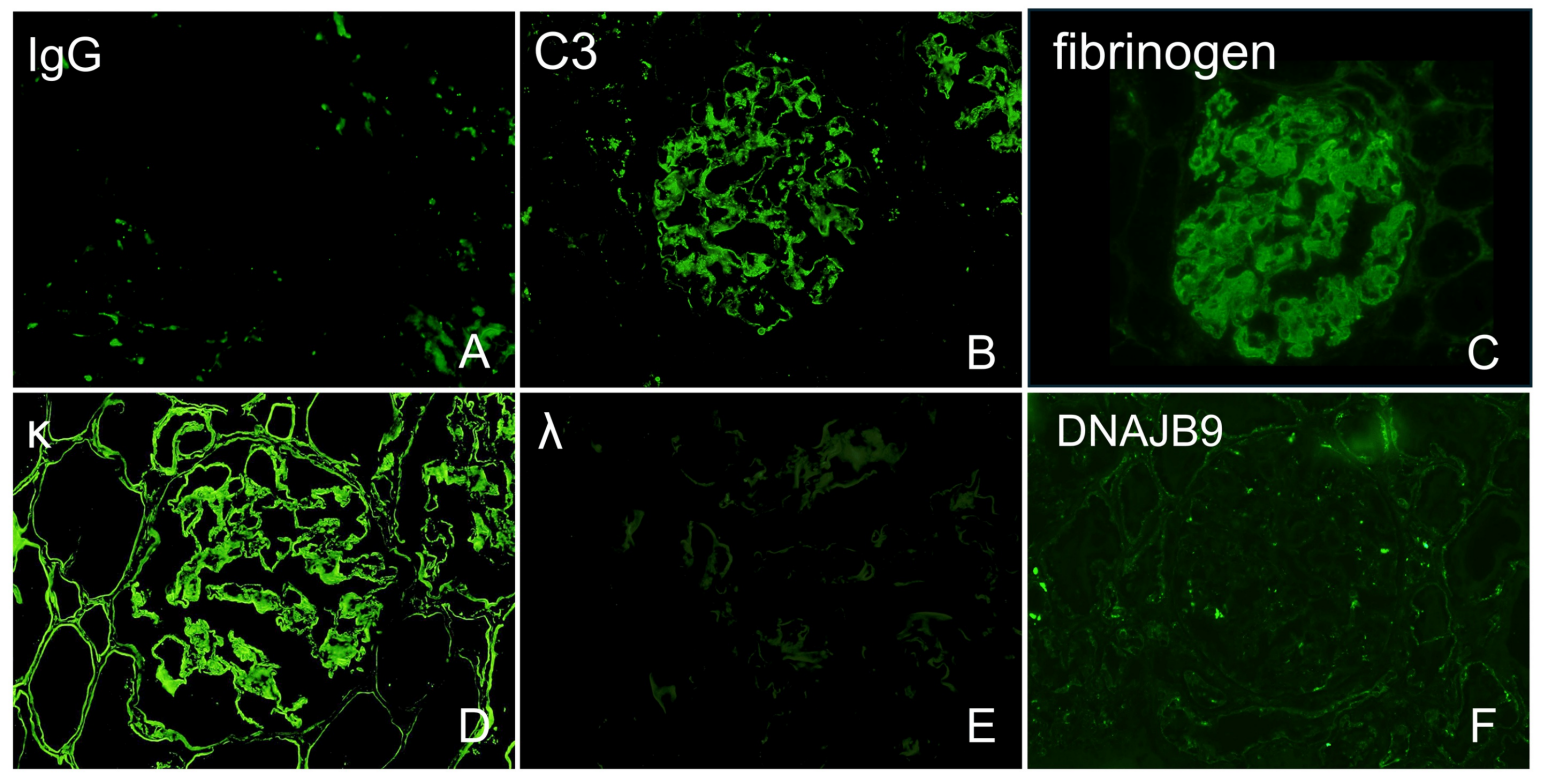
Immunofluorescence findings. By immunofluorescence, positive staining for C3, κ light chain, and fibrinogen was observed in the glomerular capillary loops and mesangium. κ light chain staining was also detected in Bowman’s capsule and the tubular basement membrane. Negative staining was observed for IgA, IgM, IgG, C4, C1q, λ light chain, and DNAJB9.

Immunofluorescence microscopy demonstrated strong κ light chain staining along the glomerular capillary walls, in the mesangial areas, Bowman’s capsule, and tubular basement membranes. C3 and fibrinogen were positive only in the glomeruli, while staining for IgG, IgA, IgM, C4, C1q, λ light chains, and DNAJB9 was negative (Figure 2).

**Figure 3:**
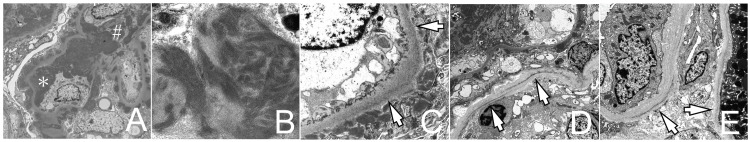
Electron microscopic findings. (A) Electron micrograph showing subendothelial (asterisk), subepithelial (arrows), and mesangial (#) deposits. (B) The electron-dense deposits are composed of thick bundles of fibrils, approximately 10 nm in diameter, arranged in a random orientation. (C–E) Powdery deposits are observed within the glomerular basement membrane (C, arrows), Bowman’s capsule (D, arrows), and tubular basement membrane (E, arrows).

Electron microscopy revealed electron-dense deposits in the mesangial areas and in the subepithelial and subendothelial regions of the glomerular basement membrane (Figure 3A). These deposits contained fibrils approximately 10 nm in diameter, forming thick, randomly oriented bundles (Figure 3B). Additionally, powdery deposits were observed in the glomerular basement membrane, tubular basement membranes, and Bowman’s capsule (Figures 3C-3E).

Taken together, the renal pathology demonstrated features characteristic of both PGNMID-LC and LCDD. Given that these lesions were attributed to monoclonal free κ light chain deposition, bone marrow aspiration was performed to evaluate for multiple myeloma. The bone marrow showed an increased proportion of monoclonal plasma cells at 30.6%, fulfilling the diagnostic criteria for multiple myeloma.

**Figure 4:**
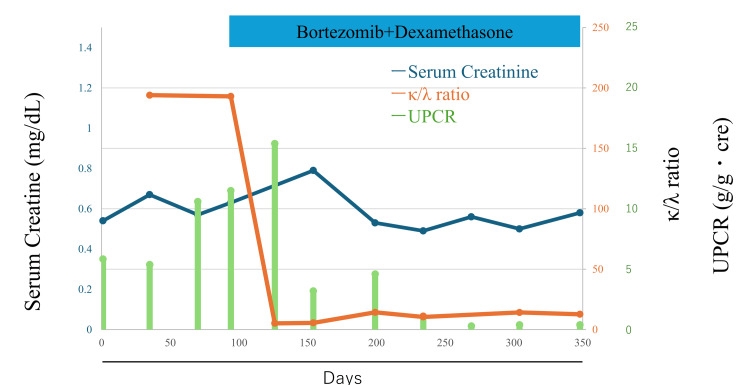
Clinical course of the patient. Trends in serum creatinine (blue line) and κ/λ ratio (orange line), and changes in urine protein-to-creatinine ratio (UPCR, green bars) over time.

The patient was diagnosed with renal involvement secondary to multiple myeloma and was transferred to a specialized hematology center for treatment. There, she was started on bortezomib-dexamethasone (BD) therapy. After approximately six months of treatment, the κ/λ ratio decreased to 12.7, and the urine protein-to-creatinine ratio improved to 0.3 g/g·creatinine (Figure 4).

## Discussion

In the present case, the presence of monoclonal free κ light chains in both the serum and urine, along with their deposition in renal tissue as demonstrated by immunofluorescence microscopy, suggested renal impairment caused by the deposition of monoclonal free κ light chains. Renal lesions associated with monoclonal immunoglobulins are classified into several types based on their pathological features [5]. Among these, conditions characterized by light chain-only deposits include LCDD, PGNMID-LC, amyloid light chain (AL) amyloidosis, light chain proximal tubulopathy (LCPT), and cast nephropathy. These are considered distinct disease entities. Of these, the three conditions that present with glomerular lesions are LCDD, PGNMID-LC, and AL amyloidosis. We herein discuss the similarities and differences between these entities and our case.

LCDD was first described by Randall et al. in 1976 and is characterized by nodular glomerular lesions and the deposition of monoclonal free light chains in the GBM and TBM [3]. However, various reports indicate that nodular glomerulosclerosis, although characteristic, is observed in only approximately 53% of cases and is not invariably present [6]. On the other hand, the features of PGNMID-LC, a subtype of proliferative glomerulonephritis with monoclonal immunoglobulin deposits (PGNMID), have been described by Nasr et al. in a case series of 17 patients [4]. According to their report, the predominant histological pattern on light microscopy was MPGN, observed in approximately 76% of cases. Immunofluorescence demonstrated deposition of either κ or λ light chains along with complement C3 in the glomeruli, but no light chain deposition was observed in the tubular basement membranes. In our case, MPGN type III-like glomerular lesions were observed, along with deposition of κ light chains and C3 in the glomeruli, findings consistent with the features of PGNMID-LC. Additionally, immunofluorescence microscopy revealed κ light chain deposition in the GBM, TBM, and Bowman’s capsule, and electron microscopy demonstrated powdery deposits in these same regions. Such findings are rarely seen in PGNMID-LC and are more characteristic of LCDD. Thus, the renal pathological findings in this case exhibited features of both PGNMID-LC and LCDD, representing a unique coexistence of characteristics from both diseases. AL amyloidosis, which is characterized by glomerular deposition of AL-type amyloid, was ruled out based on the absence of such deposits in this case.

Renal lesions caused by monoclonal immunoglobulins are classified based on the ultrastructural appearance of the deposits into two categories: glomerulopathy with organized deposits and glomerulopathy with non-organized deposits [7]. LCDD and PGNMID-LC belong to the latter group and typically do not exhibit organized structures on electron microscopy. In the present case, however, the glomerular deposits contained fibrils approximately 10 nm in diameter, which is an atypical finding. Nonetheless, cases of PGNMID-LC with fibrillar structures on electron microscopy have been reported. One such report suggested a possible association between this unusual ultrastructural appearance and local activation of the complement pathway, particularly via the alternative pathway [8]. Although our case did not demonstrate hypocomplementemia, C3 deposition was observed in the glomeruli, suggesting that complement activation may have occurred locally. Mass spectrometry is useful for analyzing the composition of deposits; however, in this case, residual renal biopsy specimens did not contain glomeruli, and analysis could not be performed. Despite this, the glomerular deposits were positive not only for κ light chains but also for fibrinogen on immunofluorescence, and PTAH staining was also positive in corresponding areas. These findings suggest that the deposits contained not only light chains but also fibrin or fibrinogen components. Taken together, the presence of fibrillar structures and the immunoreactivity for fibrin-related proteins may explain the atypical electron microscopic appearance observed in this case.

We also considered other diseases that can present with MPGN-like glomerular lesions in the differential diagnosis. These include type I cryoglobulinemic glomerulopathy, immunotactoid glomerulopathy, and fibrillary glomerulonephritis. In our case, type I cryoglobulinemic glomerulopathy was considered unlikely, as serum cryoglobulin testing was negative and electron microscopy did not reveal the fibrillary deposits with a tubular substructure characteristic of this condition. Immunotactoid glomerulopathy was also excluded based on the absence of glomerular IgG deposition and the lack of organized microtubular deposits on electron microscopy. Because fibrillar structures were observed in the glomerular deposits of our case, fibrillary glomerulonephritis was also considered in the differential diagnosis. To evaluate this possibility, we performed additional immunostaining for DNAJB9, a sensitive and specific marker for fibrillary glomerulonephritis. The result was negative (Figure 2F), and thus, fibrillary glomerulonephritis was considered unlikely [9].

As mentioned earlier, both LCDD and PGNMID-LC are believed to result from the deposition of monoclonal light chains; however, they exhibit distinct renal pathological features. The mechanisms underlying these differences remain unclear. Nasr et al. conducted a molecular analysis of light chains in patients with PGNMID-LC and reported that these light chains had different properties in the variable (V) domain compared to those found in LCDD. Notably, the light chains in PGNMID-LC were capable of activating the alternative complement pathway, which may account for the pathological differences between the two conditions [4]. However, previous reports have described cases of PGNMID-LC with light chain deposition in the tubular basement membranes and Bowman’s capsule, features more commonly associated with LCDD [10]. In addition, cases of LCDD complicated by cast nephropathy have also been documented [11]. These findings suggest that although rare, overlapping or sequential manifestations of multiple light chain-associated renal pathologies can occur in a single patient. In the present case, we speculate that a form of LCDD without nodular glomerulosclerosis may have been present initially. Over time, biochemical alterations in the monoclonal light chains may have promoted their co-deposition with fibrin or fibrinogen in the glomeruli, leading to local complement activation and the development of MPGN type III-like lesions. This hypothesis may explain the coexistence of pathological features characteristic of both LCDD and PGNMID-LC in this case. However, this remains speculative, and several important limitations must be acknowledged. First, the temporal relationship between potential LCDD and PGNMID-LC features could not be definitively established from a single biopsy timepoint. Second, molecular characterization of the light chain variable domains was not performed, which would be necessary to support the proposed evolutionary mechanism. Third, longitudinal histological data demonstrating the proposed transition from LCDD to PGNMID-LC pathology are lacking.

In cases of paraprotein-related kidney disease where multiple myeloma is diagnosed as the underlying condition, chemotherapy targeting the myeloma is typically initiated [12]. Even in patients who do not meet the diagnostic criteria for multiple myeloma, the condition is referred to as monoclonal gammopathy of renal significance (MGRS), and similar therapeutic approaches have been reported to be effective [13]. In our case, the patient met the diagnostic criteria for multiple myeloma and was therefore treated with standard chemotherapy using bortezomib and dexamethasone. The subsequent improvement in both proteinuria and renal function following therapy further supported the conclusion that the renal lesions were caused by underlying multiple myeloma (Table 2).

**Table 2:** Comparison of typical features among PGNMID-LC, LCDD, and the present case. PGNMID-LC, proliferative glomerulonephritis with monoclonal immunoglobulin deposits-light chain; LCDD, light chain deposition disease; MGUS, monoclonal gammopathy of undetermined significance; MPGN, membranoproliferative glomerulonephritis; GBM, glomerular basement membrane; TBM, tubular basement membrane.

	PGNMID-LC	LCDD	Present case
Associated hematologic disorders	• MGUS: Often no detectable hematologic malignancy	• Multiple myeloma (50-60%) • MGUS	• Multiple myeloma
M-protein type	• Monoclonal light chains. Usually, λ light chain predominant	• Monoclonal light chains. κ light chain predominant (80%)	•Monoclonal free κ light chains. No λ light chain detected
Light microscopy findings	• MPGN pattern	• Nodular sclerosis • TBM thickening	• MPGN pattern
Electron microscopy findings	• Usually non-organized deposits, rarely fibrillary structures	• Non-organized granular deposits, linear TBM deposits	• Organized fibrils (10 nm), powdery deposits
Immunofluorescence deposits	• Light chain restriction, glomerular staining, C3 positive	• Light chain restriction, linear GBM/TBM staining	• κ restriction, glomerular + TBM staining, C3 and fibrinogen positive

## Conclusions

We reported a rare case of multiple myeloma presenting with renal pathological features of PGNMID-LC, accompanied by light chain deposition in the GBM, TBM, and Bowman’s capsule, findings typically observed in LCDD. Treatment with bortezomib and dexamethasone led to improvement in both proteinuria and renal function. Although both LCDD and PGNMID-LC are caused by the deposition of monoclonal free light chains in the kidney, cases exhibiting features of both conditions simultaneously are extremely rare. The exact pathogenic mechanism remains unclear, and further case accumulation and investigation are warranted.
